# Study on natural frequency response characteristics of coal vibration excited by simple harmonic wave

**DOI:** 10.1038/s41598-022-19110-x

**Published:** 2022-09-01

**Authors:** Zhihui Wen, Libo Zhang, Jianping Wei, Jianwei Wang, Junzhao Zhang, Yannan Jia, Yongjie Ren

**Affiliations:** 1 State Key Laboratory Cultivation Base for Gas Geology and Gas Control, Henan Polytechnic University, Jiaozuo, 454000 China; 2 Collaborative Innovation Center of Coal Work Safety and Clean High Efficiency Utilization, Jiaozuo, 454000 China; 3 College of Safety Science and Engineering, Henan Polytechnic University, Jiaozuo, 454000 China; 4 Zhengzhou Coal Industry (Group) Co. LTD, Zhengzhou, 450000 China; 5 Shandong University Communist Youth League Committee, Jinan, 250013 China

**Keywords:** Energy science and technology, Fossil fuels

## Abstract

The natural frequency of coal is one of the important technical parameters for the application of the permeability enhancement technology of coal and rock forced vibration. Aiming at exploring the dominant frequency of the permeability enhancement technology of coal vibration excited by vibration wave, the model of coal vibration excited by simple harmonic wave (SHW) was constructed. Furthermore, considering the three main control parameters, i.e., excitation force, coal sample size and mechanical parameters, the response characteristics of coal vibration excited by SHW were simulated and calculated. The calculation results demonstrate that when the frequency of excitation force equals the natural frequency of coal, the vibration occurs and the peak values of response parameters all increase significantly. The peak acceleration response of coal increases with the increase of excitation force, whereas it decreases with the increase of coal size. Under the same SHW excitation force, the mechanical parameters of coal determine the vibration response characteristics of coal, and the natural frequency of coal is proportional to the elastic modulus. Finally, the variation law of natural frequency response characteristics of coal vibration excited by SHW was verified by the response experiment on coal vibration under SHW excitation and related test results. The research results can serve as a theoretical basis for the application of the permeability enhancement technology of coal vibration excited by vibration wave.

## Introduction

Vibration, including earthquake, ultrasonic wave and electromagnetic wave, is very common in nature^1–3^. Considering its damage effect, people always strive to eliminate vibration in production^4–9^. In fact, the damage effect of vibration can also be utilized in many fields^10–12^. For example, in petroleum engineering, mechanical waves generated by vibration can enhance formation permeability, thus improving oil recovery^13–18^. In coal mining, a new technical concept of using vibration wave to excite coal to improve gas extraction rate, i.e., the permeability enhancement technology of coal vibration excited by vibration wave (hereafter referred to as the CVEVW permeability enhancement technology), was proposed on the basis of the vibration oil recovery technology.

The CVEVW permeability enhancement technology can promote the development of coal fractures and improve the permeability of coal seams^19–23^. When the frequency of vibration wave is the same as the natural frequency of coal, the coal experiences vibration-induced excitation which results in fracture development and permeability enhancement^24^. At the same time, the “throwing effect” and “thermal effect” produced by vibration-induced excitation are conducive to the desorption of adsorbed gas in coal^25–27^, thus improving the adsorption/desorption characteristics of coal seam gas. Therefore, the key to realizing the CVEVW permeability enhancement technology lies in the determination of the natural frequency of coal.

In the field of engineering vibration, the natural frequency of a structure can be obtained by experiments or finite element calculation. In the experiment, the “hammering method” is usually adopted for testing the natural frequencies of coal samples. The principle is to utilize the sensor to measure the response signals stimulated by the force the hammer which instantaneously knocks on the sample^28^. By using the “hammering method”, Ren et al.^29^ measured the first-order and second-order natural frequencies of coal and rock, which are 11.1–17.2 Hz and 35.0–40.6 Hz, respectively. Li et al.^30^ also used this method to determine the initial natural frequency of coal and rock, which is 49.7 Hz. Moreover, through a white noise scanning test, they concluded that the vibration leads to the decrease of the natural frequency of coal and rock. Zhang et al.^31^ used the “hammering method” to determine the acceleration response characteristics of raw coal samples before and after being dried. They pointed out that the increase of water content often brings about a decrease in the stiffness of coal and rock, an increase in the damping ratio and a significant decrease in natural frequency.

Relevant scholars calculated the natural frequency by the finite element software. Yin^32^ analyzed the natural frequency of the rock model under different stress conditions with the aid of the finite element analysis software, concluding that its first-order natural frequency ranges from 17 to 21 kHz. With the help of the numerical analysis software, Li et al.^23^ found out that the natural frequency of rock decreases due to the existence of pores and cracks.

Proving the change characteristics of coal body natural frequency and its influencing factors is the basis of the CVEVW permeability enhancement technology. Despite extensive studies on the natural frequency of coal and rock all over the world, the size of the natural frequency of coal remains controversial. The characteristics and influencing factors of coal body vibration response are not yet clear, and the existing research results are not fully applicable to the vibration wave excitation coal body resonance infiltration enhancement technology.

Based on the theories and methods of vibration mechanics and structural dynamics, the model of coal vibration excited by simple harmonic wave (SHW) was established in this paper. Furthermore, the response of coal vibration excited by SHW was analyzed with the aid of the ANSYS finite element software. On this basis, the natural frequency characteristics of coal vibration and its influencing factors were explored.

## Coal vibration model under SHW excitation

**Figure 1:**
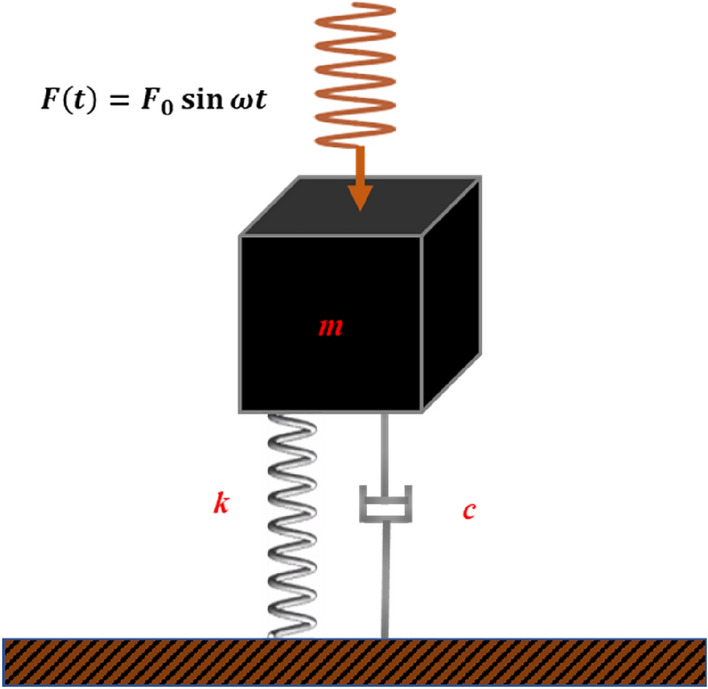
Model of coal vibration excited by SHW.

Due to its under-damped condition, coal stops vibration quickly in the absence of continuous disturbance. Under the action of external SHW excitation, the vibration propagates in the coal body in the form of harmonic waves in coal. Assuming that external excitation is a harmonic force with constant amplitude, the vibration system of coal can be represented by the physical model in Fig. 1.

When coal is subjected to continuous SHW excitation force, the differential equation of forced vibration is obtained according to Newton’s law of motion:

1 $$m\frac{{dx^{2} }}{dt} + c\frac{dx}{{dt}} + kx = F_{0} \sin \omega t$$

where *m* is the mass of coal, kg; *c* is the coal damping, dimensionless; *k* is the coal stiffness, N · m^−1^; *x* is the displacement of coal relative to the equilibrium position, m; *F*_*0*_ is the amplitude of the excitation force, N; *ω* is the frequency of the excitation force, Hz.

All solutions of Eq. (1) are composed of the general solution *x*_1_ of the homogeneous equation and the special solution *x*_2_ of the non-homogeneous equation:

2 $${\text{x}}({\text{t}})= {\text{ x}}\_\{ 1\} \,({\text{t}}) + {\text{ x}}\_\{ 2\} \,({\text{t}})$$

3 $$\left\{ {\begin{array}{*{20}l} {x_{1} \left( t \right) = e^{{ - \xi \omega_{n} t}} Bsin\left( {\omega_{d} t + \varphi } \right)} \hfill \\ {x_{2} \left( t \right) = \frac{{A_{0} }}{{\sqrt {\left[ {1 - \left( \lambda \right)^{2} } \right]^{2} + \left( {2\xi \lambda } \right)^{2} } }}sin\left( {\omega t - \theta } \right)} \hfill \\ \end{array} } \right.$$

The expressions of each physical quantity in Eq. (3) are as follows:

4 $$\left\{ {\begin{array}{*{20}l} {A_{0} = {{F_{0} } \mathord{\left/ {\vphantom {{F_{0} } k}} \right. \kern-\nulldelimiterspace} k}} \hfill \\ {\lambda = \frac{\omega }{{\omega_{n} }}} \hfill \\ {\theta = tg^{ - 1} \frac{2\xi \lambda }{{1 - \lambda^{2} }}} \hfill \\ \end{array} } \right.$$

where *A*_0_ is the static displacement of coal under the SHW excitation force, m; *B* is the amplitude of undamped coal in the ideal state, m/s^2^; λ is the frequency ratio, dimensionless; *θ* is the phase difference of the forced vibration response, rad; *ξ* is the damping ratio, dimensionless; *ω*_*n*_ and *ω*_*d*_ are the undamped natural frequency and underdamped natural frequency, respectively, Hz; φ is the phase angle, rad.

In Eq. (3), the general solution *x*_1_, which is referred as the transient response, represents the vibration whose amplitude decays exponentially; the special solution *x*_2_, which is referred as the steady-state response, represents the steady-state solution of coal displacement response under SHW excitation. It can be seen that under the action of SHW, the steady-state response of coal remains simple harmonic vibration. Besides, the frequency of forced vibration of coal is the same as that of excitation force. In addition, the amplitude of the steady-state response of coal can be expressed by:

5 $$A = \frac{{A_{0} }}{{\sqrt {\left[ {1 - \left( \lambda \right)^{2} } \right]^{2} + \left( {2\xi \lambda } \right)^{2} } }} = \frac{{{{F_{0} } \mathord{\left/ {\vphantom {{F_{0} } k}} \right. \kern-\nulldelimiterspace} k}}}{{\sqrt {\left[ {1 - \left( {{\omega \mathord{\left/ {\vphantom {\omega {\omega_{n} }}} \right. \kern-\nulldelimiterspace} {\omega_{n} }}} \right)^{2} } \right]^{2} + \left[ {2\xi \left( {{\omega \mathord{\left/ {\vphantom {\omega {\omega_{n} }}} \right. \kern-\nulldelimiterspace} {\omega_{n} }}} \right)\lambda } \right]^{2} } }}$$

According to Eq. (5), the factors affecting the amplitude of the steady-state response include the static displacement *A*_*0*_, frequency ratio *λ* and properties (such as damping ratio *ξ* and undamped natural frequency *ω*_*n*_) of coal. *A*_*0*_ reflects the influence of the SHW excitation force on the steady-state response. That is, the amplitude *A* of the steady-state response is proportional to the amplitude *F*_*0*_ of the excitation force.

In order to analyze the influence of the frequency ratio *λ* and the damping ratio *ξ* on the amplitude of the steady-state response, it is necessary to introduce a dimensionless amplitude amplification factor *β*. On this basis, Eq. (5) can be rewritten as:

6 $$\beta = \frac{A}{{A_{0} }} = \frac{1}{{\sqrt {\left[ {1 - \lambda^{2} } \right]^{2} + \left[ {2\xi \lambda } \right]^{2} } }}$$

**Figure 2:**
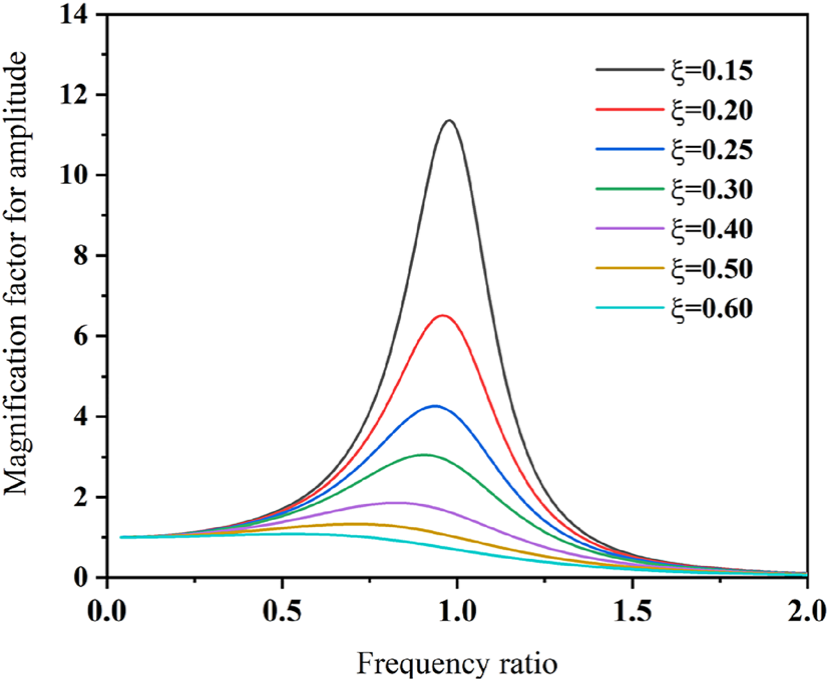
Amplitude-frequency characteristic curve of steady-state response of coal vibration.

According to the relevant vibration theory^33,34^ and Eq. (6), the amplitude-frequency characteristic curve of coal can be made with the aid of Matlab (Fig. 2).

Combined with Eq. (6), the following conclusions can be drawn from Fig. 2.

When the frequency ratio λ is far smaller than 1, that is, when the excitation frequency is far lower than the natural frequency of coal, the amplification factor *β* is close to 1.

7 $$\beta = \frac{A}{{A_{0} }} \approx 1$$

Then, the response amplitude of coal is:

8 $$A \approx A_{0} = \frac{{F_{0} }}{k}$$

Equation (8) approves that when the excitation force with a lower frequency is applied to coal, the amplitude *A* of the steady-state response is approximately equal to the static displacement under the action of the force amplitude *F*_*0*_ of the SHW excitation force^35,36^.

1. When the frequency ratio *λ* is much larger than 1, that is, when the excitation frequency is much higher than the natural frequency of coal, the amplification factor *β* is close to 0.
  9 $$\beta = \frac{A}{{A_{0} }} \approx 0$$

Then, the response amplitude of coal is:

10 $$A \approx \frac{{F_{0} }}{{k\lambda^{2} }} = \frac{{F_{0} }}{{m\omega^{2} }}$$

Equation (10) shows that when coal is excited by high-frequency SHW, the amplitude of the steady-state response *A* is mainly determined by the mass of coal. In this case, since the excitation force features high frequency and changes direction quickly, coal fails to respond to high-frequency excitation due to its own inertia^35,36^.

(2) When the frequency ratio λ is close to 1, that is, when the excitation frequency is very close to the natural frequency of coal, the amplification factor *β* reaches the maximum value.
  11 $$\beta = \frac{A}{{A_{0} }} = \frac{1}{2\xi }$$

Then, the response amplitude of coal is:

12 $$A = \frac{{A_{0} }}{2\xi } = \frac{{F_{0} /k}}{2\xi }$$

It can be observed from Eq. (12) that when the excitation frequency is close to the natural frequency, the amplitude of the steady-state response of the forced vibration surges to several times or dozens of times of the excitation force amplitude *F*_*0*_. This situation is defined as resonance, where the only limiting factor of the amplitude is damping. If there exists no damping in the vibration system, the amplitude of the vibration system will increase infinitely with time. For under-damped coal, the influence of the damping ratio ξ on the response amplitude is more obvious within this frequency range.

The forced vibration model of coal demonstrates that the steady-state response of coal under SHW excitation is still simple harmonic vibration. Besides, the frequency of vibration is consistent with that of the excitation force. The amplitude of the steady-state response will not become too high, irrespective of low or high excitation frequency. Only when the excitation frequency is close to the natural frequency, the vibration of coal is accompanied by a rapid increase in the amplitude of the vibration response. On this basis, the natural frequency of coal can be obtained through determining the corresponding frequency of the peak in the response curve, which is achieved under the SHW within a certain frequency range.

## Numerical simulation

In order to explore the characteristics of coal vibration excited by SHW, the harmonic response analysis was carried out with the aid of the ANSYS Workbench finite element software. The purpose of the analysis is to calculate the response characteristics of coal under different excitation frequencies, including displacement response, velocity response and acceleration response. It is also aimed at determining the natural frequency of coal through the peak values of displacement response, velocity response and acceleration response. When the frequency of the excitation force is close to the natural frequency, the coal will reach resonance, followed by a surge of the peak value of each parameter.

**Figure 3:**
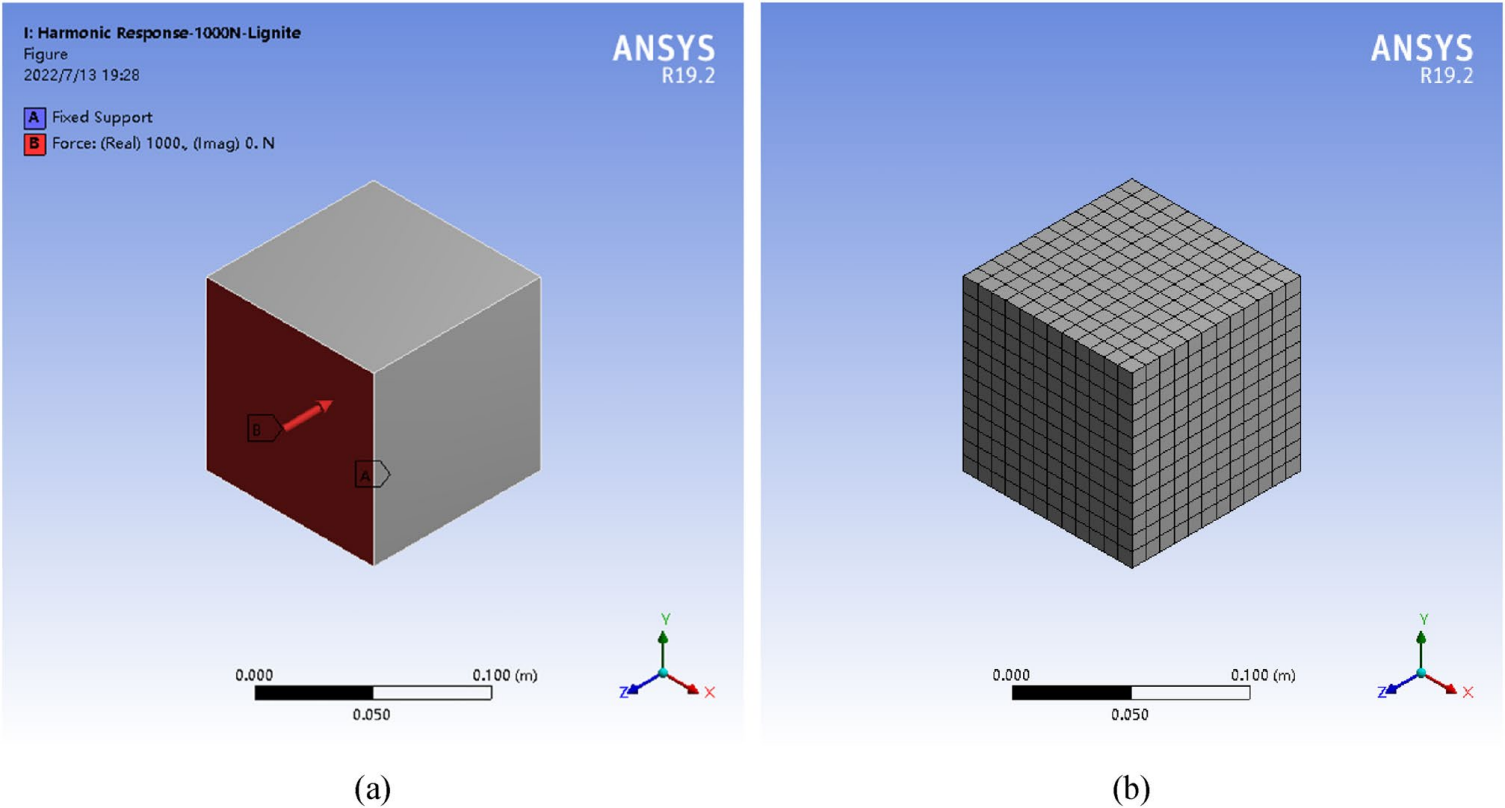
Geometric model and boundary conditions.

The numerical model of coal was square in the numerical simulation. The physical and mechanical parameters of coal model are exhibited in Table  1. In addition, the boundary conditions of the coal model are defined as follows:

1. Fixed constraints were imposed on the bottom of the square coal model;
2. The SHW excitation force in the range of 0–200 Hz was applied to one circumferential side (Fig. 3a). The coal model after meshing is displayed in Fig. 3b.

**Table 1:** Basic physical parameters of coal body.

Coal	Density *ρ* (kg/m^3^)	Elasticity modulus *E* (GPa)	Poisson’s ratio *μ*
Lignite	1120	1.11	0.32
Bituminous coal	1290	3.16	0.29
Anthracite	1380	2.67	0.30

**Figure 4:**
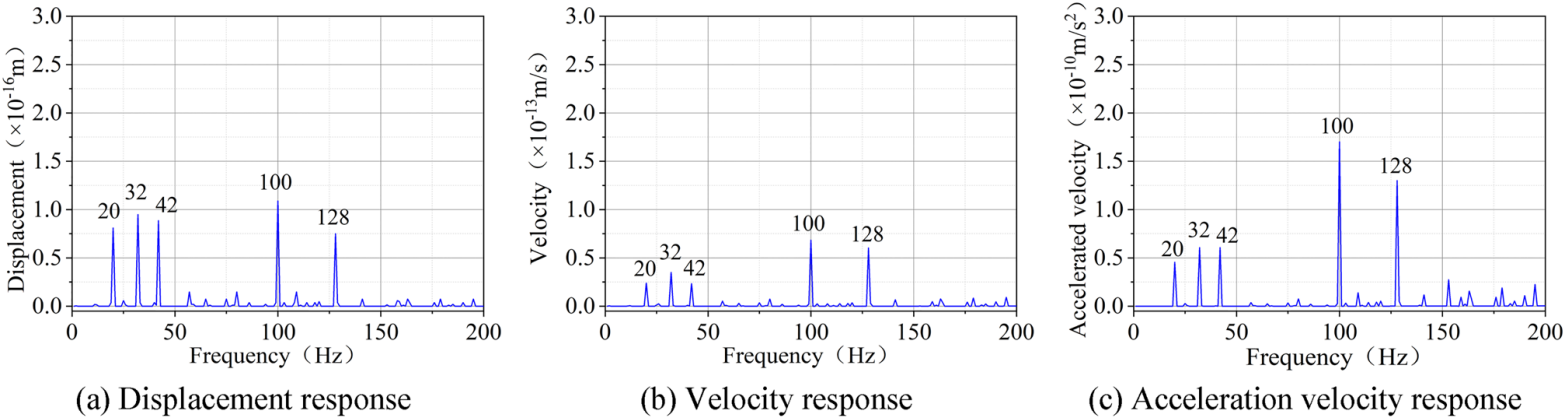
Vibration response of lignite.

Based on the harmonic response analysis carried out by the ANSYS Workbench finite element software, the displacement response, velocity response and acceleration response of coal can be obtained. Figure 4 illustrates the harmonic response analysis results of the cube lignite model with the size of 100 mm × 100 mm × 100 mm under the excitation force of 1000 N.

It can be seen from Fig. 4 that:

1. Displacement response, velocity response and acceleration response all feature five obvious peaks, and their corresponding frequencies are 20 Hz, 32 Hz, 42 Hz, 100 Hz and 128 Hz, respectively.
2. Under the same excitation force, the frequencies corresponding to the peaks of displacement response, velocity response and acceleration response are consistent. So, the natural frequency of coal can be obtained by displacement response, velocity response and acceleration response.
3. As displayed through the comparison, the ‘burr’ phenomenon is rarely observed and the five peaks are relatively obvious in the acceleration response curve.

Therefore, the study on the characteristics of coal vibration excited by SHW only requires the solution of the acceleration response curve of coal. On this basis, the correlation analysis on the influences of excitation force, coal size and mechanical parameters on the response curve should be explored.

### Magnitude of the excitation force

**Figure 5:**
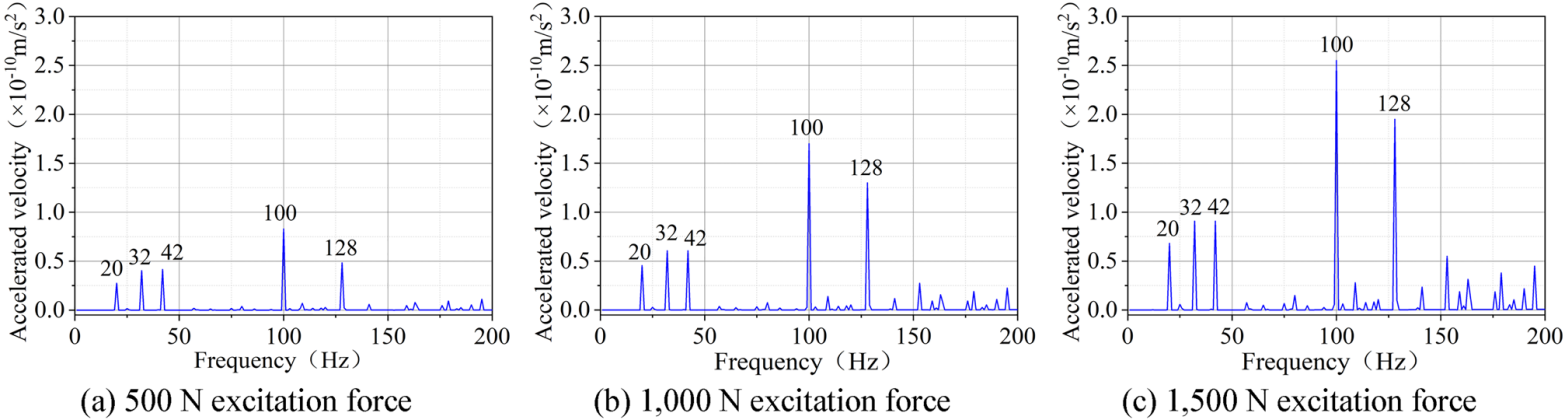
Acceleration response under different excitation forces.

When analyzing harmonic response, the magnitude of SHW excitation force exerts a profound influence on the simulation results. In this paper, with the excitation force set to 500 N, 1,500 N and 1000 N in the harmonic response analysis, the corresponding results of the square lignite model with the size of 100 mm × 100 mm × 100 mm are obtained (Fig. 5).

The following findings can be obtained from Fig. 5:

1. The acceleration response curve has five obvious peaks. Owing to the nature of resonance, only when the excitation frequency is the same as the natural frequency, an obvious peak value will appear in the acceleration response curve. Therefore, the frequencies corresponding to the five peaks are the first-order, second-order, third-order, forth-order and fifth-order natural frequencies of coal.
2. The frequency corresponding to the peak of the acceleration response of coal vibration is constant, indicating that the natural frequency is the inherent attribute of coal, which cannot be altered by the magnitude of the excitation force.
3. The peak value of the acceleration response represents the vibration intensity of coal to a certain extent. As displayed through the comparison between the peak values of the acceleration response under two excitation forces (500 N 1000 N and 1500 N), the peak value of acceleration under the former one is greater than that under the latter one. This suggests that the peak value of coal vibration response increases with the increase of the excitation force.

The above theoretical analysis shows that the amplitude of the steady-state response of the forced vibration is closely related to the amplitude of the excitation force and proportional to the magnitude of the excitation force. It is notable that the simulation results are consistent with the conclusions of the theoretical analysis.

### Size of coal

**Figure 6:**
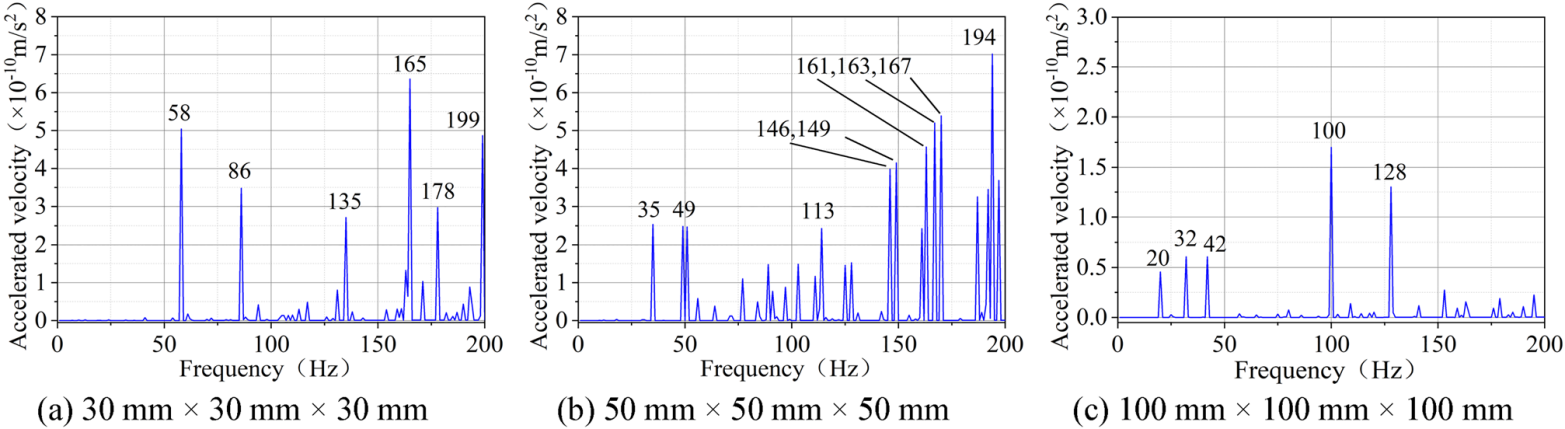
Acceleration responses of lignite models with different sizes.

Relevant studies have revealed that the mechanical properties of coal are characterized by the size effect^37–39^. Zhou^40^ found that the rock shows size effect and anisotropy as a result of joints, which become less obvious with the increase of the size. Therefore, in the harmonic response analysis simulation, the numerical models of lignite with three sizes of 30 mm × 30 mm × 30 mm, 50 mm × 50 mm × 50 mm and 100 mm × 100 mm × 100 mm were established in the hope of exploring the influence of the size of coal on the vibration response characteristics. Figure 6 gives the numerical model of cubic lignite models with size of 30 mm × 30 mm × 30 mm, 50 mm × 50 mm × 50 mm and 100 mm × 100 mm × 100 mm and their corresponding acceleration response curves under the SHW excitation force of 1000 N.

The following conclusions can be drawn from Fig. 6:

1. For coal models with different sizes, under the same SHW excitation force, the acceleration responses of coal are different. In terms of the number of the peak, the coal model with the size of 50 mm × 50 mm × 50 mm has more acceleration response peaks, which indicates that the coal of this size is more susceptible to resonance within the excitation frequency range of 0–200 Hz. In terms of the value of the peak, the larger the size of the coal is, the smaller the acceleration response peak value is. This is because the coal with the larger size needs a greater excitation force.
2. For coal models with different sizes, the frequencies corresponding to the acceleration response peaks are also different. As displayed in Fig. 6, the first two natural frequencies of the 30 mm × 30 mm × 30 mm coal model are 58 Hz and 86 Hz, respectively. The first two natural frequencies of the 50 mm × 50 mm × 50 mm coal model are 35 Hz and 49 Hz, respectively. The first two natural frequencies of the 100 mm × 100 mm × 100 mm coal model are 20 Hz and 32 Hz, respectively. Overall, the larger the coal size is, the lower the natural frequency is.

When the vibration system is a single-degree-of-freedom system, the natural frequency of the structure is proportional to the stiffness and inversely proportional to the mass^29,30^. In a nutshell, for coal with same mechanical parameters, coal with a larger size corresponds to a greater mass and a lower natural frequency.

### Mechanical parameters of coal

The matrix and minerals in coal differ greatly in terms of their mechanical properties. For example, the Young’ s modulus of organic components is about 2 GPa, whereas that of minerals is generally higher than 10 GPa^41^. Therefore, the macroscopic physical and mechanical properties of coal vary with the increase of coal rank.

**Figure 7:**
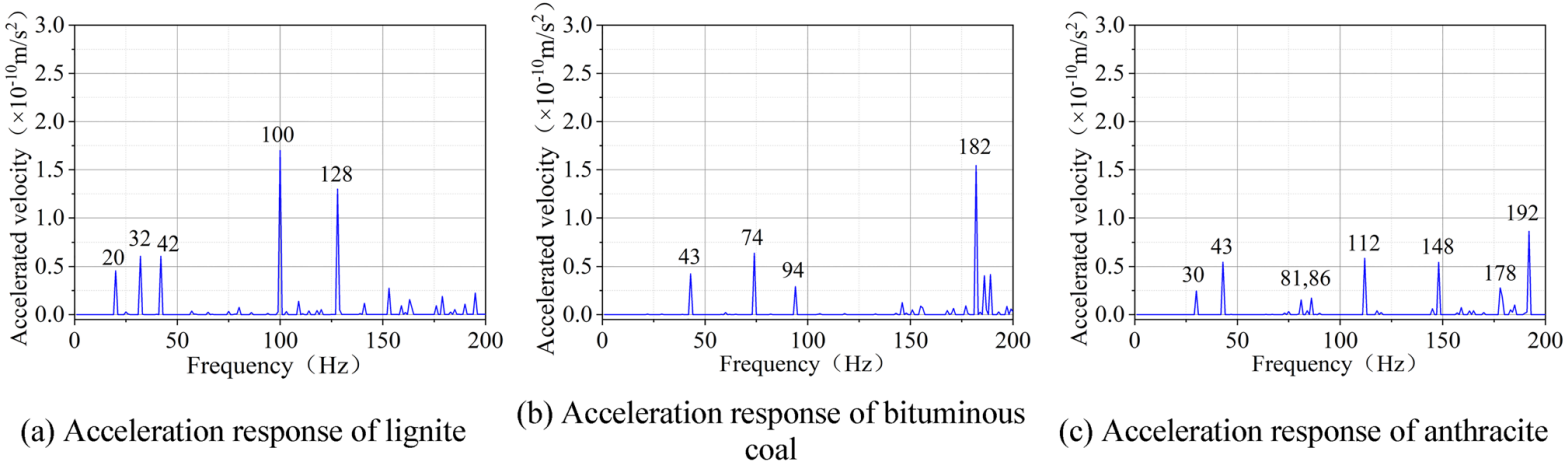
Acceleration response of coal body with different coal rank.

In order to clarify the influence of mechanical parameters on the vibration characteristics of coal, three typical coal samples of lignite, bituminous coal and anthracite were selected, and then their relevant mechanical parameters were measured (Table 1). Furthermore, 100 mm × 100 mm × 100 mm square numerical models were established for the harmonic response analysis. Based on the analysis results, the response curves of different-rank coals under the excitation force of 1000 N and the excitation frequency of 0–200 Hz were obtained (Fig. 7).

It can be observed from Fig. 7 that the first-order and second-order natural frequencies of lignite are 20 Hz and 32 Hz, respectively; those of anthracite are 30 Hz and 43 Hz, respectively; those of bituminous coal are 43 Hz and 74 Hz, respectively. The first-order and second-order natural frequencies of bituminous coal are significantly higher than those of anthracite and lignite, and those of lignite are the lowest.

Compared with the basic physical parameters of coal in Table 1, the elastic modulus of bituminous coal is the largest, followed by that of anthracite, and that of lignite is the smallest. It implies that under the same excitation force, the acceleration response and elastic modulus of coal with the same size show high consistency despite their coal ranks, which is in good agreement with the simulation results in reference^7^.

When it comes to the number of the peak, fewer peaks in discrete distribution are observed in the acceleration response of bituminous coal, while more peaks in intensive distribution are observed in that of lignite. This is because under the same SHW excitation force, the greater the elastic modulus of coal is, the more difficult the deformation of coal is. Consequently, the vibration response of coal becomes less obvious.

## Experiment on the response of coal vibration excited by SHW

**Figure 8:**
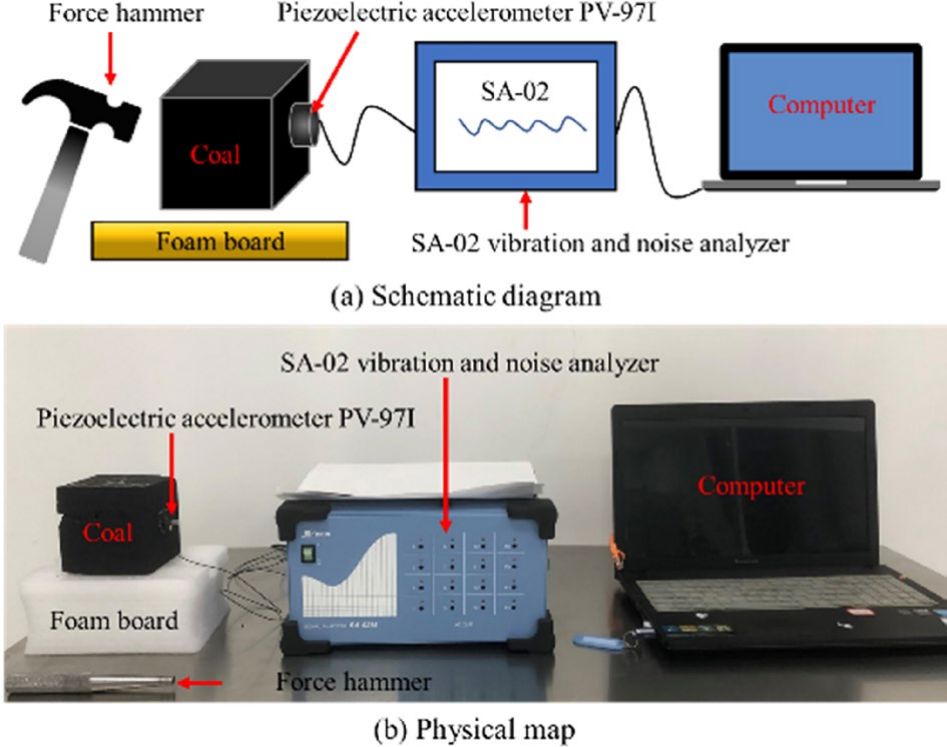
Coal natural frequency testing experimental system.

In order to further verify and clarify the response characteristics of the natural frequency of coal vibration excited by SHW, three coal samples with different mechanical properties, i.e., lignite, bituminous coal and anthracite, were selected in this study and they were cut and polished into square coal blocks of 100 × 100 × 100 mm. The natural frequency experiment was performed on different coals with the aid of the self-built coal natural frequency testing experimental system. The principle of the experimental system and the physical diagram are illustrated in Fig. 8. Experimental equipment mainly consists of a hammer, a coal sample, a foam board, a piezoelectric acceleration sensor, a vibration parameter detection and analysis device and a computer.

The specific parameters of the acceleration sensor and the vibration parameter detection and analysis device used in the experiment are shown in the literature^29^. Due to the low natural frequency of coal, the sampling frequency of the experiment was set to 200 Hz and the number of sampling points was 1024.

The natural frequency of coal was tested according to the test principle of the ‘type’. The experimental procedure is as follows:

1. Cleaning the surface of the cube coal sample and making the coal sample lie flat on the foam board. Using the foam board support for the test to avoid the influence of the external medium on the vibration parameters of the coal body;
2. The acceleration sensor was installed on the surface of the cubic coal sample by Vaseline paste;
3. Once knocked by the hammer, the coal sample generated free vibration instantaneously. At this moment, using the piezoelectric effect of quartz crystal, the piezoelectric accelerometer PV-91I collected the acceleration signals by measuring the inertia force on the coal sample, which then was analyzed and processed by the data acquisition software.
4. The SA-02 vibration and noise analyzer performs Fourier analysis on the received time-domain waveform, and the analysis results are displayed on the computer used in conjunction with the vibration parameter detection device to obtain a frequency-domain diagram.
5. In the frequency domain signal, it can be observed that the acceleration amplitude increases rapidly at a certain frequency, and the corresponding frequency is the natural frequency of the sample.

The directional differences of the mechanical properties of coal are attributed to its complex structure and typical bedding structure. Due to the directional distribution of each microstructure in coal, the uniaxial compressive strength and peak strength parallel to the bedding surface are higher than those of the vertical bedding surface. It is also responsible for the differences between the natural frequencies of coal in different bedding directions.

**Figure 9:**
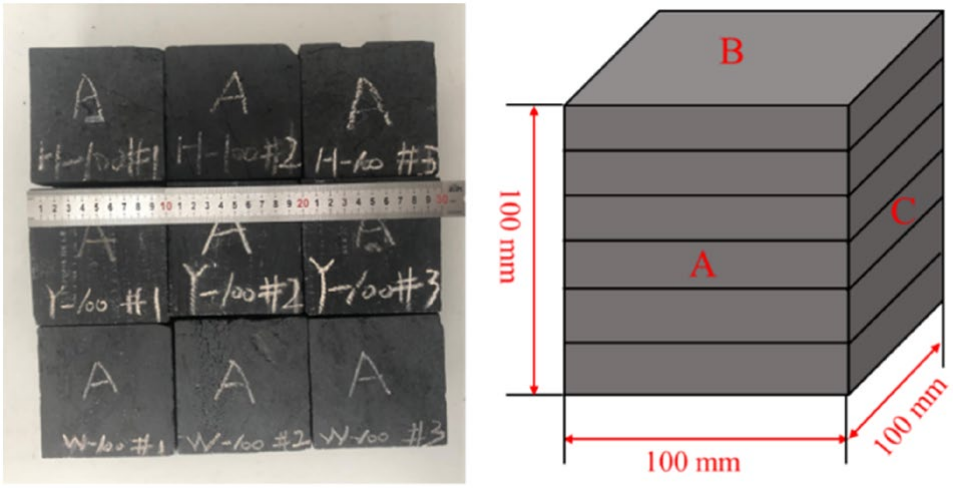
Schematic diagram of experimental coal sample and coal sample.

In order to avoid the experimental deviation caused by the different directions of coal bedding and meanwhile to make the experiment meet the Maxwell’s principle of reciprocity, the parallel bedding surface in the adjacent three surfaces of the square coal sample was marked as Surface B, and the two surfaces of the vertical bedding were marked as Surface A and Surface C, respectively. As shown in Fig. 9, the experiment was carried out on the three sides of each coal sample for 20 times.

**Figure 10:**
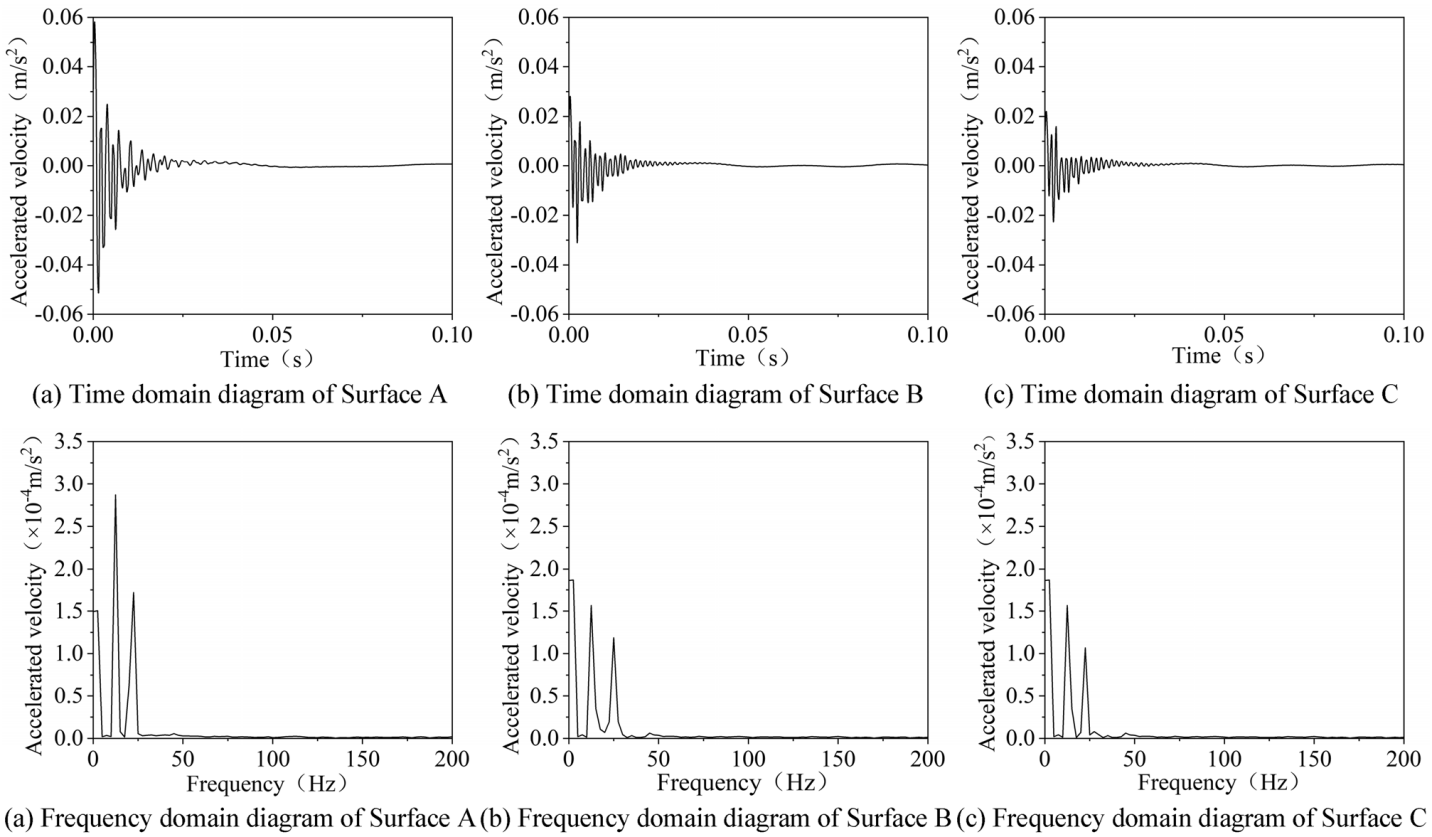
Frequency domain diagram and time domain diagram of natural frequency test on lignite No. 1 coal sample (H1).

Figure 10 displays the time domain diagram and frequency domain diagram of typical vibration signals of lignite No. 1 coal sample (H1). Through the acquisition and analysis of vibration signals, the natural frequency of the sample can be determined in the frequency domain diagram.

The abnormal signals in the experimental process are eliminated from all the experimental results. Based on the combination of the analysis on the experimental results and the test results of lignite No. 1 coal sample (H1) in Fig. 10, the first-order and second-order natural frequencies of Surfaces A and C of lignite No. 1 coal sample (H1) in all the 20 tests are 12.5 Hz and 22.5 Hz, and those of Surface B in 19 tests out of all the 20 tests are determined as 15 Hz and 25 Hz, respectively. The test results of other coal samples are exhibited in Table 2.

**Table 2:** Natural frequency test results.

Serial number	Inherent frequency/Hz								
	Surface A		Frequency of occurrence	Surface B		Frequency of occurrence	Surface C		Frequency of occurrence
	First order	Second order		First order	Second order		First order	Second order	
H1	12.5	22.5	20	15.0	25.0	19	12.5	22.5	20
H2	12.5	22.5	19	15.0	25.0	19	12.5	22.5	19
H3	10.0	20.0	20	15.0	25.0	20	12.5	22.5	20
Y1	25.0	42.5	17	32.5	45.0	18	27.5	42.5	18
Y2	27.5	42.5	20	32.5	45.0	20	27.5	42.5	20
Y3	27.5	42.5	20	32.5	45.0	20	25.0	42.5	20
W1	20.0	32.5	19	25.0	35.0	18	20.0	32.5	19
W2	20.0	32.5	20	25.0	35.0	20	20.0	32.5	20
W3	17.5	32.5	18	25.0	35.0	18	20.0	32.5	19

It can be seen from Table 2 that the measured natural frequencies are all between 10 to 50 Hz. The natural frequency of coal is its own property, and the natural frequencies of coal samples are limited by their own structure, size, shape and other factors. The natural frequency of coal body measured in previous research is mostly 0 to 100 Hz, so the measured results are in line with the general law of natural frequency of coal body. In 20 tests, the natural frequency test result of coal body is basically unchanged, indicating that the same size of raw coal The samples have good self-similarity, and the measurement error caused by different samples is small.

## Experimental results and analysis

**Figure 11:**
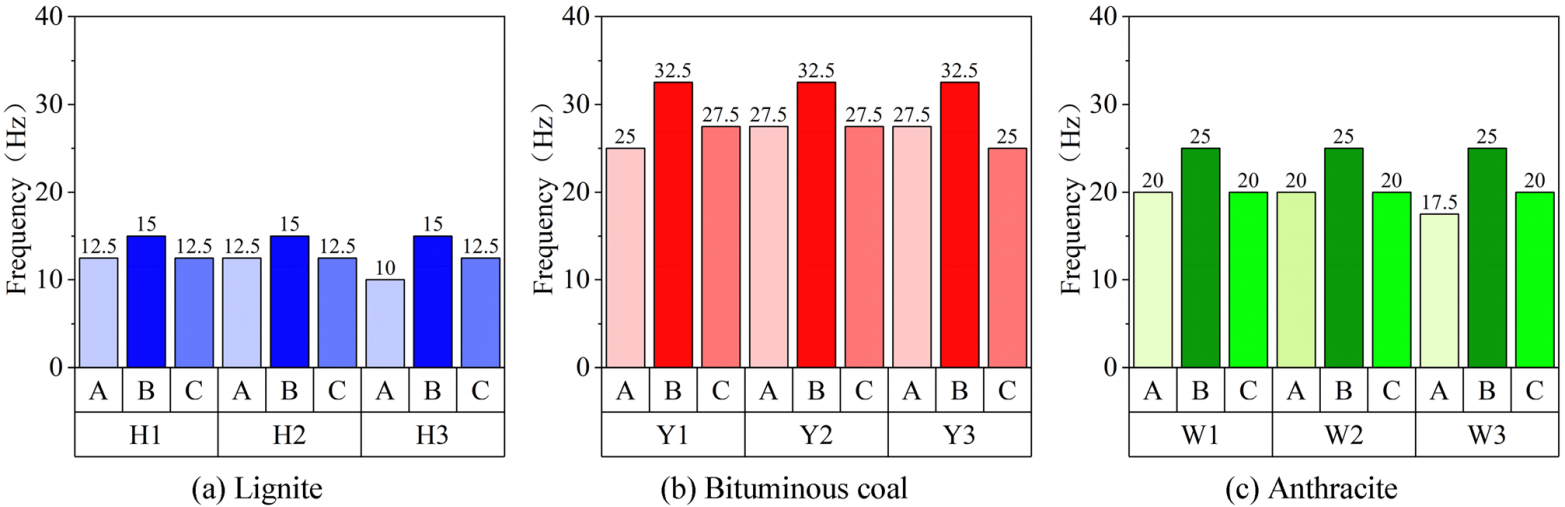
Comparison of first-order natural frequencies of three-dimensional surfaces of experimental coal samples.

In engineering applications, from the energy point of view, the low-order natural frequency of the specimen is more prone to excitation. Therefore, according to the experimental data in Table 2, the histogram of first-order natural frequencies of three-dimensional surfaces of coal samples are displayed in Fig. 11.

As displayed by the comparative analysis of Fig. 11, the first-order natural frequencies of Surfaces A, B and C of three different-rank coal samples are consistent. To be specific, for the three lignite samples, the first-order natural frequencies of Surface A range from 10 to 12.5 Hz; those of Surface B are all 15 Hz; and those of Surface C are 12.5 Hz. For the bituminous coal samples, the first-order natural frequencies of Surface A range from 25 to 27.5 Hz; those of Surface B are 32.5 Hz; and those of Surface C are in the range from 25 to 27.5 Hz. For the anthracite samples, the first-order natural frequencies of Surface A are in the range from 17.5 to 20 Hz; those of Surface B are 25 Hz; and those of Surface C are 20 Hz. The following conclusion can be drawn: For the three typical coal samples with high, medium and low coal ranks, lignite has the lowest natural frequency, followed by anthracite, bituminous coal has the highest natural frequency. Besides, the natural frequencies of three surfaces of bituminous coal are all higher than those of lignite and anthracite.

Comparing the first-order natural frequencies of the three surfaces of coal samples A, B, and C, it can be seen that the first-order natural frequency of surface B is the highest. The directional distribution characteristics of the various microstructures in the coal body lead to regular changes in the mechanical properties of the coal samples. The uniaxial compressive strength and peak strength parallel to the bedding plane are greater than those of the vertical bedding plane^42^. The B plane is parallel to the bedding plane. Therefore, the first-order natural frequencies of all coal faces B are higher than those of the remaining faces.

With respect to the relevant mechanical parameters of the three different-rank coal samples in Table 1, the elastic modulus of bituminous coal is the largest, followed by that of anthracite, and that of lignite is the smallest, which coincide with the variation of natural frequency. Such results indicate that the larger the elastic modulus is, the higher the natural frequency is, which is in good agreement with the above simulation results.

## Conclusion

In this paper, the model of coal vibration excited by SHW was constructed, and then the response analysis of coal vibration excited by SHW was carried out with the aid of the ANSYS finite element software. The numerical simulation results were verified by relevant experiments. Furthermore, the response characteristics of the natural frequency of coal vibration excited by SHW were explored. The following conclusions were drawn:

1. Under the excitation by SHW, the steady-state response of coal vibration still belongs to harmonic vibration. Besides, the frequency of the steady-state response of coal is consistent with that of the excitation force. When the excitation frequency is close to the natural frequency, the amplitude of forced vibration response increases rapidly. In this case, the only limiting factor of amplitude is damping.
2. The frequencies corresponding to the peak values of acceleration response are the different-order natural frequencies of coal. The magnitude of excitation force does not alter the natural frequency. The peak value of acceleration response represents the vibration intensity of coal, and it increases with the increase of the excitation force.
3. Under the same SHW excitation force, the larger the coal size is, the smaller the peak acceleration response is. This is because the excitation of larger-sized coal vibration requires a greater force.
4. The simulation and experimental results both demonstrate that the natural frequency and vibration characteristics of coal are affected by its mechanical parameters. Under the same SHW excitation force, the coal with a larger elastic modulus is less likely to deform. Consequently, its vibration response is less obvious.

## Data Availability

The data used to support the findings of this study are included within the article.
